# Multiple Object Tracking Using the Shortest Path Faster Association Algorithm

**DOI:** 10.1155/2014/481719

**Published:** 2014-08-17

**Authors:** Zhenghao Xi, Heping Liu, Huaping Liu, Bin Yang

**Affiliations:** ^1^School of Automation and Electrical Engineering, University of Science and Technology Beijing, Beijing 100083, China; ^2^State Key Laboratory of Intelligent Technology and Systems, Tsinghua University, Beijing 100084, China; ^3^Department of Electronic Engineering, Changwon National University, Changwon 641-773, Republic of Korea

## Abstract

To solve the persistently multiple object tracking in cluttered environments, this paper presents a novel tracking association approach based on the shortest path faster algorithm. First, the multiple object tracking is formulated as an integer programming problem of the flow network. Then we relax the integer programming to a standard linear programming problem. Therefore, the global optimum can be quickly obtained using the shortest path faster algorithm. The proposed method avoids the difficulties of integer programming, and it has a lower worst-case complexity than competing methods but better robustness and tracking accuracy in complex environments. Simulation results show that the proposed algorithm takes less time than other state-of-the-art methods and can operate in real time.

## 1. Introduction 

Multiple object tracking is a hot topic in the field of computer vision. Robust tracking of objects is important for many computer vision applications, such as human-computer interaction, video surveillance, intelligent navigation [1, 2]. Apart from a high performance detection algorithm as an auxiliary, high quality multiobject tracking should also track the algorithm for support, which can address certain types of complex cases, for example, illumination, occlusion, clutter, and so on [3]. The data association (DA) method is a favorite for multiobject tracking. The often utilized techniques include the nearest neighbor method [4], joint probability data association [5], and methods based on neural networks [6].

The effect of the DA methods mentioned above is closely related to the detection accuracy in adjacent frames. These typical approaches are resilient to false negatives and false positives: if an object is not detected in a frame but is in previous and following frames, it is a false negative. A false positive is mistaking the tracking object “A” as object “B.” Although this problem can be solved using targeted design a statistical trajectory model with filtering [7, 8], the calculation method that provides the maximum posterior probability is NP-complete.

Recent papers have proposed different approaches to this problem. Giebel et al. [9] use sampling and particle filtering to remove clutter from the same object and reduce the probability of NP-completeness. This method obtains a relatively accurate tracking trajectory but requires a sufficient number of sampling points. Perera et al. [10] divides a long sequence into several short ones, yielding lots of short tracking tracks, and links them using Kalman filtering. This can avoid the NP-completeness. The accuracy of this method is inversely proportional to the length of the short tracking tracks, the shorter the length, the better the tracking. However, the excessive division increases the computation time, due to which the method cannot track objects for long time. Fleuret et al. [11] processes trajectories individually over long sequences using reasonable greedy dynamic programming (DP) to choose the order. These approaches, while effective, cannot attain the global optimal solution.

Zhang's approach [12] relies on a min-cost network flow framework-based optimization method to find the global optimum for multiple object tracking. However, the two algorithms he proposes have several defects in practice and their complexity is polynomial. Under this framework, Berclaz et al. [13] formulate multiobject tracking as an integer programming (IP) problem and reduce it to linear programming (LP). By relying on the k-shortest paths (KSP) algorithm for the optimization of the LP problem, their approach reduces the complexity to perform robust multiobject tracking in real time. However, because of KSP's lack of a motion model over dynamic programming (DP), the tendency of the DP to ignore fragmentary trajectories makes it more robust. Pirsiavash [14] continues the work of Zhang and uses his method to obtain the global optimal solution with the greedy algorithm for *K* = 1 in *O*(*N*) but only obtains the approximate solutions for *K* > 1 in *O*(*KN*), where *K* is the unknown optimal number of unique tracks.

By contrast, in this paper, we effectively combine the models proposed by Zhang and Berclaz to devise a more efficient framework for the shortest path faster algorithm (SPFA). Not only can the SPFA algorithm directly obtain the global solution, it also shows the advantage of the DP motion model, which enables the algorithm to ignore incomplete trajectories and behave more robustly against this type of noise. Moreover, it is far better with respect to both the worst-case complexity and the run time than the above-mentioned state-of-the-art algorithms. Our main contributions in this paper are as follows.

1. Based on the min-cost network model, we introduce a novel general mathematical integer programming formulation for multiobject tracking. The proposed IP method is conducive to naturally filtering out false positives and false negatives using SPFA.
2. To solve the integer linear programming formulation of the proposed framework and to obtain the global optimal solution, we propose to use the more rapid and more efficient SPFA algorithm. Compared with the state-of-the-art methods of [13, 14], the SPFA algorithm can improve the running time obviously while the multiobject tracking precision and accuracy are not loss.

The rest of this paper is organized as follows. In Section 2, we formulate an IP using the min-cost network flow framework and relax it to continuous LP.  Section 3 contains our proposed shortest path faster algorithm for the relaxation of the original IP. We introduce approaches to target localization and long sequence segmentation processing in Section 4.  Section 5 contains the experimental results and a complete evaluation metrics. Finally, conclusions are drawn in Section 6.

## 2. Network Flow Framework

The target motion of multiobjet tracking can be better described using the relationship between the neighborhood locations that use the DP method in a min-cost network flow framework. We define an objective function for multiobject tracking in the same manner as in [13]. The objective presence of likelihood will be estimated by the marginal posterior probability in every frame, thereby obtaining the potential trajectory of the moving object.

### 2.1. Min-Cost Flow Model

We formulate the multiobject tracking as a process, where the objective location of each object discretely changes in continuous time. A directed 3D spatiotemporal group with random variable *k*_*t*_ is used to describe the video sequence. Consider

(1) $$\begin{array}{c} k_{t}=\left(x,y,t\right),\;k_{t}\in V, \end{array}$$

where *k*_*t*_ denotes any location of an object in this spatiotemporal group at time *t*, *V* is the set of all space-time locations in a sequence, and *x* and *y* are the pixel positions of the target in the transverse and longitudinal axes, respectively.

For any location *k*_*t*_ at time *t*, the neighborhood *N*(*k*_*t*_) ⊂ {1,2,…, *K*} denotes the locations that an object can reach at time *t* + 1. A single track as an ordered set of state vectors *T* = (*k*_1_,…, *k*_*N*_), and *X* = (*T*_1_,…, *T*_*L*_) is a set of tracks. We assume that the tracking tracks are independent of each other and describe the network flow framework of multiobject tracking using the dynamic model as follows:

(2) P(X)=∏T∈XP(T),where  P(T)=Psource(k1)(∏n=1N−1P(kn+1 ∣ kn))Psink(kN).

*P*_source_(*k*_1_) is the probability of a tracking track starting at location *k*_1_ and *P*_sink_(*k*_*N*_) is the probability of a tracking track ending at location *k*_*N*_.

In the spatial coordinate set *V*, a binary indicator variable *φ*_*i*,*t*_ represents the directed flow from location *k*_*i*_ to location *k*_*t*_; that is, it stands for the number of objects moving from *k*_*i*_ to *k*_*t*_. *φ*_*i*,*t*_ is 1 when the space-time locations *k*_*i*_ and *k*_*t*_ are included in some track, given that the object is at *k*_*t*−1_ at time *t*, which means that an object remains at the same spatial location between times *t* − 1 and *t*. For locations *k*_*t*_ and *k*_*j*_ at time *t* + 1, some constraint conditions are executed for the variable *φ*_*i*,*t*_:

(3) $$\begin{array}{c} \forall k_{t},\;\underset{k_{i},k_{t}\in N\left(k_{i}\right)}{\sum }\varphi_{i,t}=\underset{k_{j}\in N\left(k_{t}\right)}{\sum }\varphi_{t,j}, \end{array}$$

(4) $$\begin{array}{c} \forall k_{i},k_{t},\;\underset{k_{t}\in N\left(k_{i}\right)}{\sum }\varphi_{i,t}\leq 1. \end{array}$$

Let a random variable *M*_*t*_ stand for the true presence of an object at location *k*_*t*_ in space time. For every instant of time *t*, the detector is used to check every location of the tracking zone. The marginal posterior probability of an existing object is calculated as follows:

(5) $$\begin{array}{c} \rho_{t}=\hat{P}\left(M_{t}=1\;|\;I_{t}\right), \end{array}$$

where *I*_*t*_ is the single image at frame *t*. We write *m* = {*m*_*t*_} for a feasible set of the likelihood probability distributions for the existence objects in *V* by the method in Section 4.1. *M* is the spatial set of *M*_*t*_. The likelihood probability of the existence of an object in the given set of tracks *X* is

(6) $$\begin{array}{c} P\left(M=m\;|\;X\right)=\underset{k_{t}\in X}{\prod }P\left(M_{t}=m_{t}\;|\;X\right). \end{array}$$

*M*_*t*_ is conditional independence in *X*. We can infer the maximum posteriori estimate of tracks by the probability distributions of the existence of objects:

(7)  X∗=argmaxXP(X)P(M=m ∣ X)

(8)   =argmaxX∏T∈XP(T)∏kt∈XP(Mt=mt ∣ X)

(9)   =argmaxX∑T∈Xlog⁡P(T)+∑kt∈Xlog⁡P(Mt=mt ∣ X)

(10)  =argmaxX∑T∈Xlog⁡P(T)+∑kt[(1−mt)log⁡P(Mt=0 ∣ X) +mtlog⁡P(Mt=1 ∣ X)]

(11)  =argmaxX∑T∈Xlog⁡P(T)+∑ktmtlog⁡P(Mt=1 ∣ X)P(Mt=0 ∣ X)

(12)  =argmaxX∑T∈Xlog⁡P(T)+∑ktmtlog⁡(ρt1−ρt),

where (10) is true because *m*_*t*_ is 0 or 1 according to (4), and (11) is obtained by ignoring a term that does not depend on *m*_*t*_. The cost value of a directed flow between the neighborhood locations of any adjacent frames is defined as

(13) $$\begin{array}{c} c\left(e_{t,t+1}\right)=-\log\left(\frac{\rho_{t}}{1-\rho_{t}}\right), \end{array}$$

where *e*_*t*,*t*+1_ is a directed edge from location *k*_*t*_ at time *t* to location *k*_*t*+1_ at time *t*+1, and the total cost between any two locations in *V* is

(14) $$\begin{array}{c} C\left(e_{i,j}\right)=\underset{\begin{array}{c} e_{t,t+1}\in e_{i,j}\\ k_{t+1}\in N\left(k_{t}\right) \end{array}}{\sum }c\left(e_{t,t+1}\right). \end{array}$$

### 2.2. Integer Linear Programming

**Figure 1:**
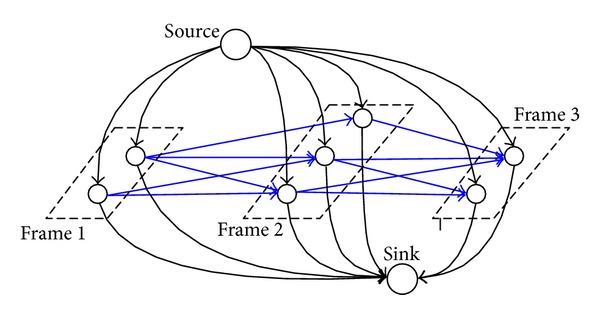
The simple flow network model.

In our framework, because the objects can enter and leave the tracking area, we introduce additional nodes for the source and sink that have been defined proposed by [13]. Equations (7)–(12) can then be translated naturally into an integer linear program (ILP):

(15) Minimize C(φ)=C(ei,j)∑kj∈N(ki)φi,j+C(esource,i) ×∑kiφsource,i+C(ei,sink)∑kiφi,sinkSubject  to ∀kt, ∑ki,kt∈N(ki)φi,t=∑kj∈N(kt)φt,j∀ki,kt, ∑kt∈N(ki)φi,t≤1,

where the constraint conditions are the same as (3) and (4), and φ* = argmin *C*(*φ*) is the optimal solution of the ILP. *C*(*e*_source,*i*_) is the total cost of the flow from the source node to the locations of the tracking track, and *C*(*e*_*i*,sink_) is that from the locations of the track to the sink node.  Figure 1 shows a simple flow network constructed from multiobject tracking, where the costs are *c*(*e*_*i*,*j*_) for blue edges, *c*(*e*_source,*i*_) and *c*(*e*_*j*,sink_) for black edges.

The costs are defined as follows:

(16) $$\begin{array}{c} c\left(e_{\text{source},i}\right)=-\log P_{\text{source}}\left(k_{i}\right),\\ c\left(e_{i,\text{sink}}\right)=-\log P_{\text{sink}}\left(k_{i}\right). \end{array}$$

The relaxation of the IP using standard methods is NP-complete. In general, the variants of the simple algorithm [15, 16] or the interior point based methods [17, 18] can be used to solve this problem. However, these algorithms have very high worst-case time complexities. In [13, 14], whereas the methods of KSP and successive shortest path (SSP) can relax the IP successfully to continuous LP, both of them have their own deficiencies. We use the SPFA algorithm to compensate the deficiencies of these methods.

## 3. Fast Dynamic Shortest Path Algorithm

In this paper, we use the shortest path faster algorithm to relax the integer program by the network flow framework; the average case complexity of this algorithm is *O*(*E*). The global optimum of the SPFA algorithm makes the tracking more reliable and more efficient. The network flow framework needs two particular properties to realize the SPFA algorithm as follows.

1. All edges and nodes are independent of each other; all edges are unit capacity.
2. The network is a directed acyclic graph (DAG).

### 3.1. SPFA Algorithm

The shortest path faster algorithm has been proposed in [19]. The data structure of the SPFA algorithm uses an adjacency list and a First-in, First-out (FIFO) queue. Applying the dynamic optimal approach, the time complexity of SPFA algorithm is *O*(*E*), where *E* is the number of edges in the graph. It is better than the complexity of Dijkstra's algorithm, *E* ≪ *N* ^2^, where *N* is the number of nodes. No particular limitation conditions are needed for this algorithm. Therefore, the SPFA algorithm can be adopted for all directed graphs, except for the ones where negative weight cycles are reachable from the source.

### 3.2. SPFA Algorithm with Virtual Nodes

Let *C* be the total cost of any location in space *V*, and let *E* be the set of the edges between adjacent frames of any neighborhood location. The state transition between any pair of nodes of the model can be attained by *E*, and the DAG *G*(*V*, *E*, *C*) can completely describe the flow activity of an object of the min-cost flow model.

In our min-cost flow model, *Q* is a FIFO queue, *L* denotes an adjacency list used to store *G*(*V*, *E*, *C*), and *c*(*e*_*i*,*j*_) is an element of *L*. Let array *D* record the current cost of a directed flow from* source* to all other nodes. The total cost value of the shortest path from the* source* to *v* is stored in array *D*(*v*). In the initialization, each element of array *D* has its maximum value. Array *D* will then output the shortest path between the* source* and the* sink* through the SPFA algorithm when queue *Q* is empty.

To improve the robustness of multiobject tracking in an environment of false negatives, we define *G*_*r*_ as the residual graph of *G*(*V*, *E*, *C*) that denotes all locations from the current node to the terminal node. Two additional virtual nodes,* source* and* sink*, are introduced into *G*_*r*_ and are linked to all nodes representing locations. We can then find the shortest path between node* source* and node* sink* by the SPFA algorithm in *G*_*r*_. Moreover, the shortest path between* source* and node *v* can be obtained in array *D*, where *v* is any node in the shortest path from the* source* to the* sink*.

In the proposed min-cost flow framework, we can obtain the shortest path through the following steps.

1. Create the FIFO queue *Q*, the adjacency list *L*, and the array *D*. Initialize *D*(*j*) : = *∞* and *D*(source) : = 0, where* source* is the beginning node and *j* is any other node. Add* source* to the queue *Q*.
2. Add all neighborhood nodes that can be reached form* source* to *Q* and record their cost values in array *D*. Let *D*(*i*) store the total cost value of the shortest path from the* source* to the node *i*, *i* ∈ *G*_*r*_.
3. Assess the neighborhood nodes *j* of the new node *i* in *Q*, where *j* is the node that can be reached from node *i*. If *D*(*i*) + *c*(*e*_*i*,*j*_) < *D*(*j*), *D*(*j*) : = *D*(*i*) + *c*(*e*_*i*,*j*_).
4. Iterate (3) until queue *Q* is empty and the shortest path *T* = (*k*_1_,…, *k*_*N*_), *k*_*N*_ ∈ *G*_*r*_, between* source* and the node *v* can be obtained in array *D*, where *v* is any node in the shortest path from* source* to* sink*.

**Figure 2:**
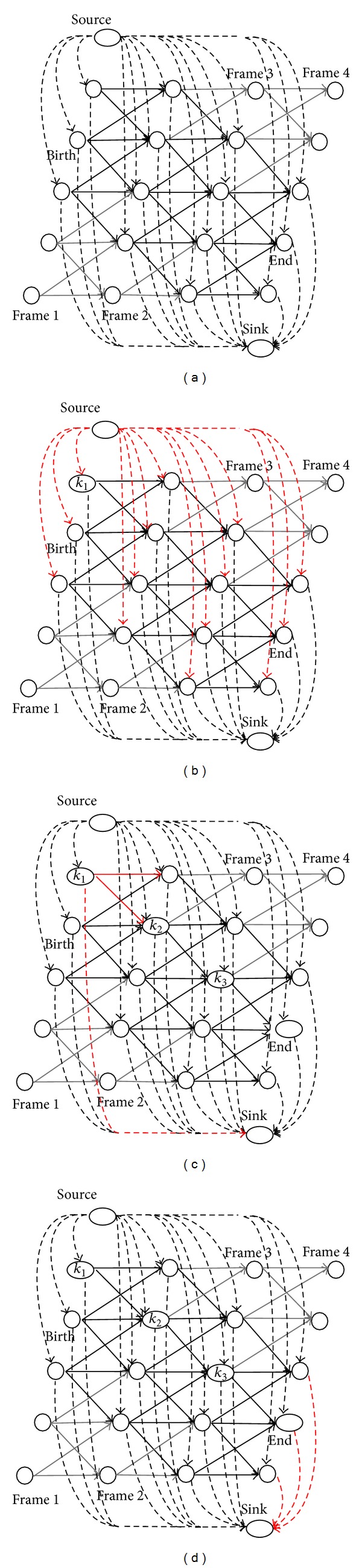
The shortest path faster algorithm. (a) Recording all nodes of *G*_*r*_ in an adjacency list, starting from the* source* node. Adding the* source* into the queue *Q*, let *D*(source) = 0; (b) adding all the nodes that can be reached from* source* to *Q* and recording the cost values in *D*. (c) Adding all nodes that can be reached from *k*_1_ to *Q* and recording their total cost values. If a node has been in queue *Q*, update its total cost value to the smaller value in *D*; (d) iterating (c) until queue *Q* is empty and the shortest path *T* = (*k*_1_,…, *k*_*N*_), *k*_*N*_ ∈ *G*_*r*_ from* source* to* sink* can be obtained at the same time. Legend: black solid line, all edges among positions that can be reached, red solid line, all edges from the current position to potential locations that can be reached, black dashed line, all edges between virtual positions and the potential locations that can be reached, and red dashed line, all edges from the current position (or virtual positions) to virtual positions (or potential locations) that can be reached.

Figure 2 shows the simple processing steps of the SPFA algorithm in our proposed model. Here,* birth* represents the node where an object was first discovered, and* end* is that where it was last discovered. Each relaxation operation using the SPFA algorithm is a process of the current node visiting adjacent nodes. The *n*th relaxation operation ensures that the path is the shortest in *n*. As the length of the edge for the shortest path in the residual graph does not exceed *N* − 1, the path that we obtain using the SPFA algorithm is the shortest one. Compared with the method in [14], which uses the SSP algorithm with the additional greedy method, the SPFA algorithm can find the global optimum. Its convergence has been proved in Theorem 2 of [19].

It is not sufficient to be able to track multiple objects by the SPFA algorithm because some target movements during this process are easily overlooked. To enable the SPFA algorithm to better describe the movement of the target, we offer additional constrains for the algorithm.

### 3.3. Constraints for SPFA Algorithm

When we search the shortest path between* birth* and* end* in the original residual graph *Gr*, one problem arise. It is that the algorithm cannot handle the entry and departure of the object in any position between* birth* and* end*; that is, the tracking process is incomplete and not robust.

To improve the tracking robustness by the SPFA algorithm, we use the neighbors of* birth* and* end* to replace the original position and form a new DAG with the virtual positions* source* and* sink*, as shown in Figure 1.* Source* and* sink* here denote the positions where an object appears and disappears, respectively. This method can optimize the dynamic correlation between the nodes of the SPFA algorithm.

Moreover, at no iteration the SPFA algorithm generates a large amount of calculation because there are only three neighborhood locations calculated in each relaxation for a node, and the number of available nodes is inversely proportional to the number of iterations.

### 3.4. Time Complexity Analysis

The Dijkstra's algorithm is recognized as an effective method to compute the shortest path in *O*(*N*log⁡*N*) time. Unfortunately, in our proposed flow network, there are negative costs, which contradict the precondition of the Dijkstra's algorithm. Fortunately, there are no negative weight cycles in the proposed model and thus the SPFA algorithm can be adopted.

The proposed algorithm is an optimization of the Bellman-Ford algorithm. While we blindly go through each edge for *N* rounds in the Bellman-ford algorithm, a queue is maintained in SPFA to make sure that we only check the relaxed nodes. SPFA is simpler than the *O*(*NE*) of the Bellman-Ford algorithm, where *N* is the number of nodes and *E* is the number of edges.

For the DAG, the average case complexity of the SPFA algorithm is *O*(*E*), where *E* is the number of edges in the graph. In this case, each node enters the queue only once. The SPFA algorithm is a breadth-first search algorithm, which is the common case in our proposed approach. If each node enters the queue *N* − 1 times, the proposed algorithm degenerates into the Bellman-Ford algorithm with a time complexity that is the worst-case complexity of that algorithm, that is, *O*(*NE*). The complexity of the SPFA algorithm in the general case has been proved in [19]. Reference [20] analyzes the theoretical and experimental worst-case complexity of the SPFA algorithm in detail.

References [13, 14] propose the KSP and SSP algorithms, respectively, to compute the relaxation of the integer linear program. The worst-case complexity of both algorithms is *O*(*KN*log⁡*N*), where *K* is the unknown optimal number of unique tracks and *N* is the frame number of the video sequence. Note that because of the different values of *K*, [14] uses different methods to obtain the solution. The specific complexity of this algorithm is related to the value of *K*.

The average case complexity of our proposed algorithm is *O*(*E*), which is far less than that of the above mentioned methods. The worst-case complexity of the SPFA algorithm is *O*(*NE*), but this almost is never obtained.

Moreover, like the KSP algorithm, the SPFA algorithm successfully calculates the global optimal solution, as proved in [19]. However, SSP with the greedy algorithm as in [14] cannot obtain the global optimal solution.

## 4. Target Localization and Long Sequence Processing

High quality multiobject tracking requires a reliable tracker, a detector that can accurately segment and locate multiple objects, and a preprocessing method that can improve the performance of the algorithm.

### 4.1. Target Detection and Localization

**Figure 3:**
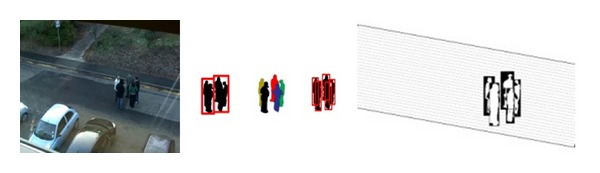
Separating merged objects and locating them with the probability distribution.

To obtain the accurate target for the tracker, we establish a background model with the improved codebook algorithm and extract the observed characteristic information of the tracking object by the foreground/background subtraction method of [21]. Using the method from [22], we segment objects that were initially merged together. We then obtain the probability distributions of the planes of the objects from the detector, and these can serve as the input to the SPFA algorithm. A few selected frames of target localization are shown in Figure 3.

Full range tracking in the camera field of view increases the processing time of the algorithm and consumes a significant portion of the limited memory resources. For this reason, because most of the calculated probabilities of the objective presence are 0, we can reduce the number of nodes and computational cost by this characteristic. On the other hand we limit the potential birth area of targets to reduce the amount of computation. The proposed method also checks the maximum detection probability of each location *k*_*t*_ within a given spatiotemporal neighborhood of each frame *t*:

(17) $$\begin{array}{c} \underset{\begin{array}{c} ||k_{\alpha }-k_{t}||<\varepsilon_{1}\\ t-\varepsilon_{2}<\alpha <t+\varepsilon_{2} \end{array}}{\max}\rho_{\alpha }. \end{array}$$

If the value at a location is below the set threshold, an object represented by the value is considered unable to reach the location, and all flows from and to it are removed from the model. This method can reduce by an order of magnitude the number of required variables and constraints. In our experiment, we pruned the graph by a radius of *ε*_1_ = *ε*_2_ = 3.

### 4.2. Long Sequence Processing

In theory, processing a long sequence using the SPFA algorithm can yield the global optimum for tracking time but requires a large amount of operation time. To address this issue, we split the long sequence into segments of 100 frames each, which yields good results with a delay of less than 0.5 seconds between input and output and can be performed in real time.

**Figure 4:**
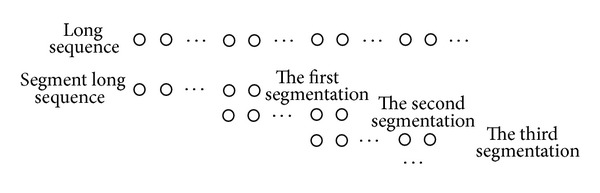
Segment processing of a long sequence.

For each segment maintaining temporal consistency, we use the method of multiframe overlay, as shown in Figure 4, and add the last 10 frames of the previously optimized segmentation to the first 10 frames of the current one.

We then force the sum of flows of every location of the first 10 frames of the current frame to be consistent with the total number of flows of the last locations of the object in the last 10 frames of the previous one. This effectively solves the problem of the missing target on the piecewise point:

(18) $$\begin{array}{c} \forall k_{t}\in \left\{1,\ldots ,K\right\},\;\underset{k_{j}\in N\left(k_{i}\right)}{\sum }\varphi_{i,j}=\underset{k_{i}\in N\left(k_{t}\right)}{\sum }\varphi_{t,i}=\theta_{t}, \end{array}$$

where *θ*_*t*_ is the total flow of the last position *k*_*t*_ of object appearing in the last 10 frames of the previous segment. For the corresponding first position *k*_*j*_ of an object appearing in the first 10 frames of the current segment, the net flow into it is equal to the flow out of position *k*_*t*_ and is also equal to the total flow out of any potential position *k*_*i*_ of any object between *k*_*t*_ and *k*_*j*_. This is implemented as an additional constraint in our model.

If we cannot find the tracking target in the first 10 frames of the current segment, the proposed method searches for the object in *t*′ frames after the current one. In our experiment, we let *t*′ = 10. If we find the tracking target in a frame within *t*′, this frame is the first frame of the current segment; the tracking fails otherwise.

## 5. Experimental Results

In our simulation, video sequences with different characteristics were selected from the PETS09, CAVIAR, BEHAVEDATA, and ETHMS (BEHAVEDATA, http://groups.inf.ed.ac.uk/vision/BEHAVEDATA/INTERACTIONS/index.html, CAVIAR, http://groups.inf.ed.ac.uk/vision/CAVIAR/CAVIARDATA1/, ETHMS, http://www.vision.ee.ethz.ch/~aess/dataset/, and PETS09, http://www.cvg.rdg.ac.uk/datasets/index.html) datasets. The challenges for each of these are summarized in Table 1. The selected sequences cover almost all problems that commonly occur in multiobject tracking.

### 5.1. Parameter Setting

In the training period, a detector is designed using the background subtraction method of the improved codebook algorithm model. We combine the detection result with the activity scope of the object by foreground/background segment update in real time and calculate the location of the object with a high probability. Because the size of the activity scope of the object and the number of the pixels of the object are not identical in every sequence, our method can generate 900–1000 detections per frame in each video sequence. We set the log-likelihood ratio of each detection process to be the negative score as the results of the linear detector.

We used a bounded value dynamic model: we define the cost *c*_*i*,*j*_ between two locations in consecutive frames in the case of spatial overlap (i.e., an object remains at a location) as 0. The costs from the virtual location to the neighborhood of* birth* and* end* are *c*_source,birth_ = 10, *c*_end,sink_ = 10, respectively. Moreover, because global search using SPFA is in the established adjacency list, finding the shortest path must be the global optimal solution without auxiliary constraints.

### 5.2. Evaluation Metrics

Let GT_*i*,*t*_ be the *i*th ground truth bounding box for the *t*th frame, and let TR_*i*,*t*_ be the tracked bounding box. *C*_*i*,*t*_ for the *t*th frame and *i*th object is defined as the ratio between the area of intersection GT_*i*,*t*_∩TR_*i*,*t*_ and the area of union GT_*i*,*t*_ ∪ TR_*i*,*t*_ [23]:

(19) $$\begin{array}{c} C_{i,t}=\frac{\text{AREA}\left\{\text{G}{\text{T}}_{i,t}\cap \text{T}{\text{R}}_{i,t}\right\}}{\text{AREA}\left\{\text{G}{\text{T}}_{i,t}\cup \text{T}{\text{R}}_{i,t}\right\}}. \end{array}$$

In our experiment, we set the threshold of *C*_*i*,*t*_ to 0.5, which means that the tracking is successful when the overlapping areas of the ground truth bounding box and tracked bounding box exceed 0.5.

**Figure 5:**
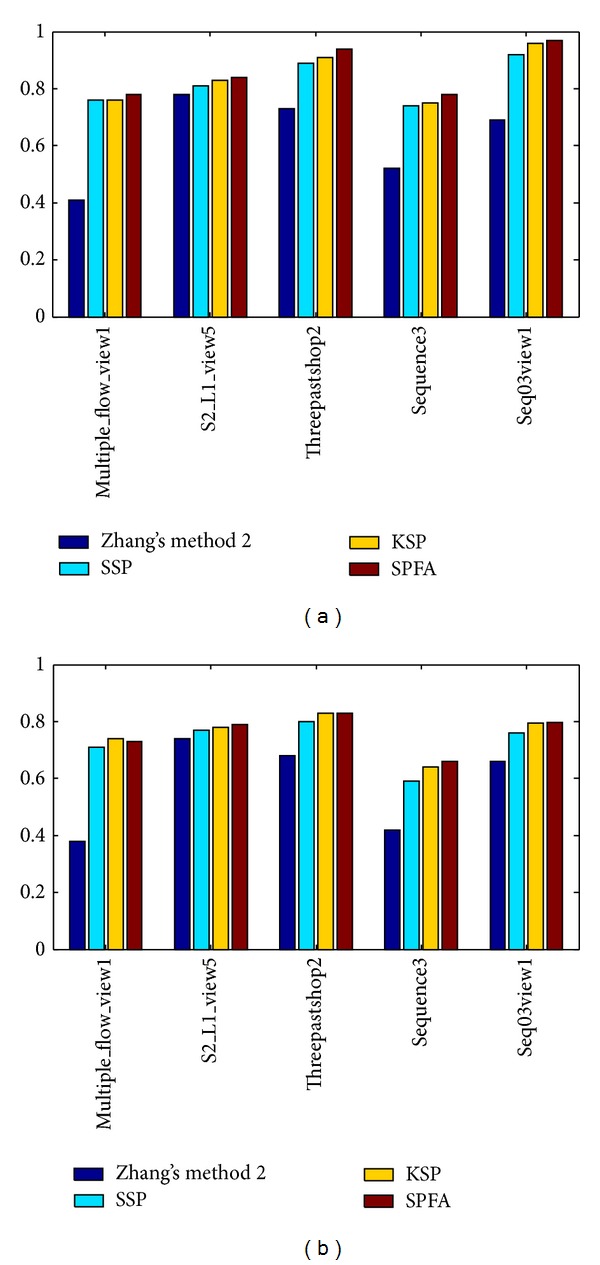
MOTA (a) and MOTP (b) measures applied to the results of recently proposed trackers (Zhang's method 2, SSP, and KSP) and our SPFA tracker on various experimental sequences.

Our results are evaluated using the multiple object tracking accuracy (MOTA) and multiple object tracking precision (MOTP) metrics of the standard CLEAR2006 metrics [24]:

(20) $$\begin{array}{c} \text{MOTA}=1-\frac{\sum_{t}\left(c_{m}\left(m_{t}\right)+c_{f}\left(fp_{t}\right)+c_{s}\right)}{\sum_{t}g_{t}}, \end{array}$$

(21) $$\begin{array}{c} \text{MOTP}=\frac{\sum_{i,t}C_{i,t}}{\sum_{t}Nm_{t}}, \end{array}$$

where *g*_*t*_ is the number of ground truth objects in the *t*th frame, *Nm*_*t*_ refers to the number of mapped objects in the *t*th frame, *m*_*t*_ represents the missed detection count, and *fp*_*t*_ is the false positive count for each frame. *c*_*s*_ = log_10_ID*-*SWITCHES_*t*_, where ID*-*SWITCHES_*t*_ is the number of ID mismatches in *t* considering the mapping in frame *t* − 1. We started the count from 1 because of the log function. *c*_*m*_ and *c*_*f*_ represent, respectively, the cost functions for missed detections and false positives. The values used for the weighting functions in (20) are *c*_*m*_ = *c*_*f*_ = 1.  Figure 5 shows the histograms of MOTA and MOTP in the experiment using the SPFA algorithm.

### 5.3. Analysis of Results

To ensure the unique identification for each tracking target, we use different colors to indicate the order. The sequences used in our experiment are from Table 1. The detection results are obtained by the process described in Section 4.1 as the input of our algorithm. We then conduct a performance test of the multiobject tracking circumstances of false positives, false negatives, and a dynamic background, respectively.

#### 5.3.1. Performance Test for False Negatives

**Figure 6:**
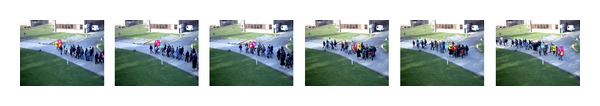
The typical results of Multiple_flow_view1 (Frame: 12, 17, 24, 48, 55, and 75).

**Figure 7:**
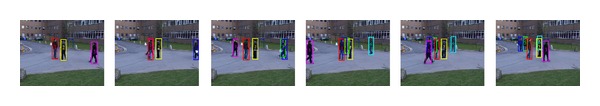
The typical results of S2_L1_view5 (Frame: 26, 50, 57, 83, 93, and 103).

The sequences use Multiple_flow_view1 and S2_L1_view5 from the PETS09 dataset. We show typical results in Figures 6 and 7. In particular, the former uses bright yellow coats worn by pedestrians as the tracking object. Although the probability of false negatives increases significantly because of occlusion with nontracking objects, the SPFA algorithm can ensure persistent tracking (the color of the tracking box has not changed) for each object in the entire tracking process. The experiment for S2_L1_view5 verifies the robustness of the SPFA algorithm when the targets leave the area of nonrestricted departure and reappear soon.

#### 5.3.2. Performance Test for False Positives

**Figure 8:**
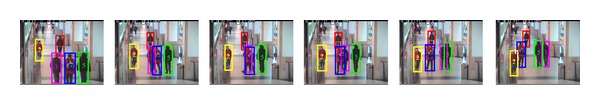
The typical results of Threepastshop2 (Frame: 375, 453, 459, 465, 484, and 509).

**Figure 9:**
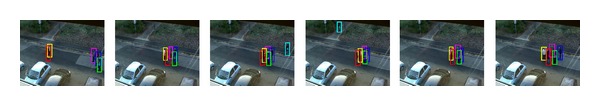
The typical results of Sequence3 (Frame: 2751, 2825, 3430, 3750, 3900, and 5010).

The sequences use the Threepastshop2 of the CAVIAR dataset and Sequence3 of the BEHAVEDATA dataset. Typical results are shown in Figures 8 and 9. We used the method from Section 4.1 for detection and localization. Because of the superior solution and anti-interference of the SPFA, we can stably track multiple objects in a timely fashion in case of false positives.

#### 5.3.3. Performance Test for Dynamic Background

There are two conditions that must be satisfied by the sequence of the experiment.

1. The available probability distribution of the dynamic background of the sequence needs to be relatively consistent. Only in this way can the algorithm quickly obtain the location of an object for tracking.
2. The targets should be fixed access areas in the tracking ground. Because the tracking ground is moving, the potential area in which the objects can enter and exit changes. We require the borders of the camera field of view to be the area for all objects that can enter and exit.

**Figure 10:**
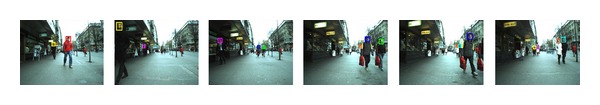
The typical results of Seq03view1 (Frame: 10, 40, 70, 100, 103, and 125).

The sequence uses Seq03view1 from the ETHMS dataset. We obtain object characteristics by the method of combining skin color and the method in [25] and show the typical results in Figure 10. The method of detection and localization in Section 4.1 only considers the available probability distribution of the target characteristic in the tracking ground and does not relate to the background conditions. Therefore, the sequence for our experiment requires a consistent probability distribution. This constraint, in a way, limits the experimental conditions of performance for a dynamic background but does not affect the conclusion that multiobject tracking using the SPFA algorithm in a dynamic background is robust.

### 5.4. Simulation Analysis

All of above experiments were performed on a Windows XP PC equipped with a 2.7 GHz Pentium (R) Dual-Core CPU and 8 GB of memory. The software platform uses Visual Studio 2010 and Open CV2.2.

**Figure 11:**
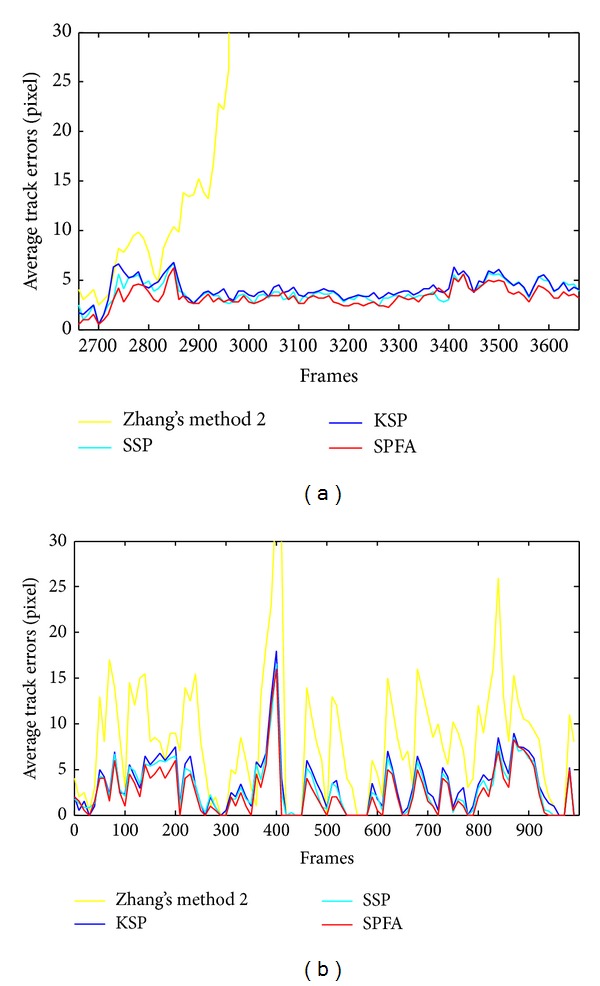
The comparison of the average tracking errors with Sequence3 of the BEHAVEDATA dataset (a) and Seq03view1 of the ETHMS dataset (b).

**Figure 12:**
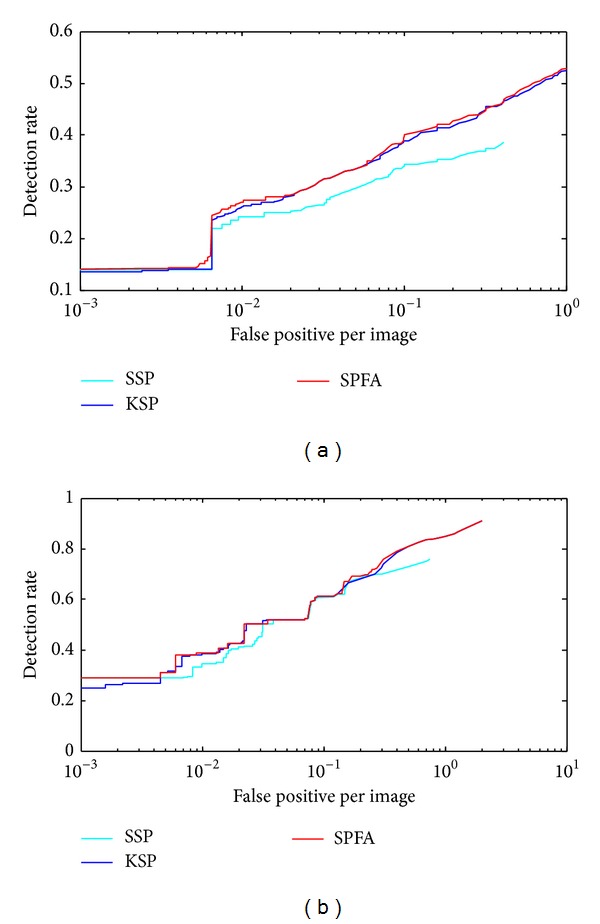
Detection rate versus false positive image on Sequence3 of the BEHAVEDATA dataset (a) and Seq03view1 of the ETHMS dataset (b).

We contrasted the SPFA algorithm with three other algorithms (Zhang's method 2 [12], KSP [13], and SSP [14]) in two sequences from different datasets (Seq03view1 of the ETHMS dataset and Sequence3 of the BEHAVEDATA dataset) with regard to the average tracking errors. The results are shown in Figure 11. We also compared the algorithms with respect to the tracking accuracy.  Figure 12 shows detection rate versus false positives per image (FPPI) for all algorithms. We use the same detection method detailed in Section 4.1 for all our experiments.

Figure 11 shows that the tracking errors of these algorithms are not significantly different in cases not involving occupancy and clutter. However, when tracking an object in the case of false positives and false negatives for a long time, our SPFA algorithm exhibits clear superiority. Although the occupancy problem in the case of simple assumptions can be satisfied by Zhang's method 2, the required assumptions result in omission and eventually lead to tracking failure when several false negatives and false positives occur frequently. In Figure 12, when the above algorithms have the same target detection rate, the SPFA algorithm performs better than other algorithms in controlling FPPI. The superiority of the SPFA algorithm is due to its faster relaxation method and to finding the global optimal solution more quickly.

**Figure 13:**
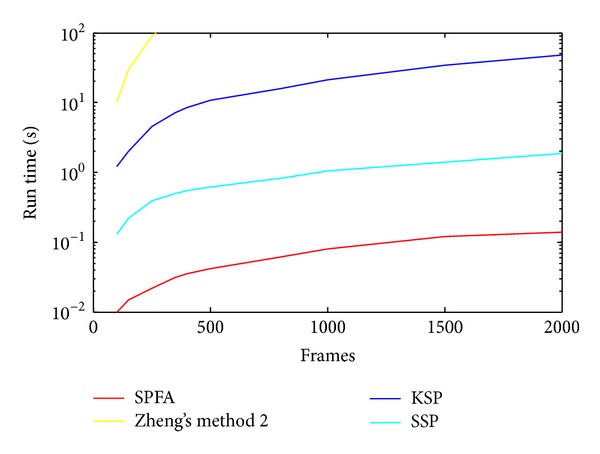
The comparison of run time.

With the same target detection method as above, we compared the false positives generated using SPFA method with those from the other methods on the ETHMS dataset and the CAVIAR dataset, as shown in Table 2. The results show that the SPFA algorithm can track better. Further, as shown in Figure 13, the run time of the SPFA algorithm significantly outperforms the other three algorithms.

### 5.5. Run Time

We evaluated the speed of our SPFA tracking algorithm on the sequences of the BEHAVEDATA dataset at 25 fps. The curves of the run time for SPFA and the above algorithms have been shown in Figure 13. The vertical axis representing run time is plotted on a log scale. The solution of Zhang's method 2 does not converge for a significant running time. When dealing with a sequence of 1000 frames, the KSP solver takes approximately 20 seconds and SSP takes 0.9 seconds, but our SPFA solver only takes 0.08 seconds.

## 6. Conclusions

In this paper, we proposed a reliable tracker with a flow network framework. In the min-cost flow model established by the theory of integer program, we then used SPFA algorithm to relax the integer assumption and to successfully identify the global optimal solution. The resulting algorithm can better solve the problems of short-time false positives and false negatives in multiobject tracking and is more robust than state-of-the-art methods. Our proposed method can quickly find the global optimal solution of the relaxed LP by using SPFA.

Experiment results indicate that the proposed algorithm is helpful in improving trajectory consistency and solving serious occlusion problems between multiple objects and can satisfy real time measurement requirements. Compared with other algorithms, there are obvious advantages of SPFA. Tracking multiple types of targets with a dynamic background in real time will be the focus of our future research.

## Figures and Tables

**Table 1:** The challenges of the experimental sequences.

Sequence name	Occ.	Scaling	Pose	Clutter	Ill	Dynamic background	Blur
Multiple_flow_view1	√			√	√		√
Threepastshop2	√	√					
Sequence3	√		√	√	√		√
S2_L1_view5	√	√	√	√	√		
Seq03_view1	√	√				√	

**Table 2:** Our algorithm's performance compared with the state-of-the-art methods for the ETHMS and CAVIAR datasets.

Dataset	Algorithm	False positives per image
ETHMS	Zhang's method 2	0.97
	KSP	0.86
	SSP	0.89
	SPFA	0.77

CAVIAR	Zhang's method 2	0.105
	KSP	0.057
	SSP	0.636
	SPFA	0.051
